# Prenatal ultrasound utilization and associated factors among pregnant women attending antenatal care in south Wollo zone public hospitals, north east, Ethiopia, 2023

**DOI:** 10.3389/fdgth.2025.1547547

**Published:** 2025-04-28

**Authors:** Belay Susu, Kibir Temesgen, Sindu Ayalew, Selam Yibeltal, Tadele Emagneneh, Adem Yesuf, Chalie Mulugeta

**Affiliations:** ^1^ Department of Midwifery, College of Health Sciences, Woldia University, Woldia, Ethiopia; ^2^ College of Medicine and Health Sciences, School of Nursing and Midwifery, Wollo University, Dessie, Ethiopia; ^3^ Department of Midwifery, School of Nursing and Midwifery, College of Medicine and Health Sciences, Wollo University, Dessie, Ethiopia

**Keywords:** antenatal care, obstetric ultrasound, prenatal, pregnant women, utilization

## Abstract

**Background:**

Prenatal ultrasound (US) is essential in antenatal care worldwide and offers significant benefits for maternal and neonatal health. It should be a standard procedure in low- income countries. However, its utilization remains poor in nations such as Ethiopia.

**Objective:**

This study aimed to assess the use of prenatal ultrasound and associated factors among pregnant women who attended antenatal care in South Wollo Zone Public Hospitals, Northeast Ethiopia, in 2023.

**Method:**

An institution-based cross-sectional study was conducted among 590 pregnant women from December 30, 2022, to February 28, 2023, in selected South Wollo Zone Public Hospitals. The data were coded, cleaned, and entered into Epi-Data version 4.6 and subsequently exported to SPSS version 26 for analysis. The strength of the association between the dependent and independent variables was presented as odds ratios (ORs) at a 95% confidence interval (95% CI), with a *P*-value of less than 0.05 according to multivariable logistic regression.

**Results:**

The prevalence of prenatal ultrasound utilization was 62.8% [95% CI: 58.7%–66.8%]. The significant factors associated with utilization included urban residency (AOR = 4.82, 95% CI: 2.99–8.03), mothers’ knowledge (AOR = 7.36, 95% CI: 4.06–13.32), educational status above primary (AOR = 2.10, 95% CI: 1.09–4.05), medical illness (AOR = 3.03, 95% CI: 1.64–5.59), government employment (AOR = 4.05, 95% CI: 1.70–9.64), and private employment (AOR = 2.34, 95% CI: 1.58–7.05).

**Conclusion:**

The proportion of patients who underwent prenatal ultrasound was lower than the WHO recommendation. The factors most significantly associated with ultrasound utilization were women's knowledge, urban residency, educational status, medical illness, and occupation. Therefore, the author recommended for health care providers educating mothers on the purposes of obstetric ultrasound and including a prenatal ultrasound screening as part of antenatal care is needed.

## 1 Introduction

Medical imaging is indispensable for medical practice today. Obstetric ultrasound is a harmless, inexpensive, and noninvasive imaging modality that helps to scan a pregnant mother's abdominal and pelvic cavity with high-frequency sound waves and delivers a real-time images of the fetus to parents (1). Diagnostic ultrasound is recognized as a safe, effective, and highly flexible imaging modality capable of providing clinically relevant information about most parts of the body rapidly and cost-effectively. Prenatal ultrasound utilization is an integral part of antenatal care worldwide (2).

The International Federation of Gynecology and Obstetrics (FIGO) recommends two ultrasound examination services for all pregnant women (3). Similarly, in 2016, the World Health Organization (WHO) recognized the benefits of offering at least one antenatal ultrasound scanning service, before 24 weeks of gestation for all pregnant women (4).

Ultrasound has been used to diagnose obstructed labor, non-cephalic presentation, single or multiple pregnancy, incomplete miscarriage, molar pregnancy, ectopic pregnancy, fetal abnormality, intrauterine growth restriction and placenta previa (5, 6). Although the degree of diagnostic accuracy may vary depending on when pregnant women present themselves for an ultrasound exam (7). Applications of ultrasound also extend to abdominal, musculoskeletal, cardiac, renal, pulmonary, trauma and soft tissue and vascular conditions (7–9).

A study shown that a minimum of three screening tests should be performed during pregnancy. The first should be performed at the fetal age of 10–14 weeks to detect abnormalities and pathological conditions in early pregnancy. The second one has to be performed between the fetal ages of 18 and 22 weeks to assess detailed fetal anatomy and rate of development. The third should be performed between the fetal age of 30 and 34 weeks to assess fetal anatomy, rate of development, placentation and circulation (10). In low-income countries where there is a lack of machines and qualified sonographers, high-risk conditions during pregnancy might be undetected until delivery. Information generated on maternal, fetal and placental conditions using ultrasound imaging enhances the diagnosis and management of life-threatening conditions (9, 10).

Ultrasound in antenatal care (ANC) is crucial for assessing maternal health, pregnancy progression and fetal development (11). In developed countries such as Vietnam and India, the prevalence of ultrasound scans during ANC is high, averaging 6.6 scans per 8 visits (12). Another study conducted in Vietnam revealed that, women scans 8–10 times throughout pregnancy (13). In low- and middle-income countries (LMICs), the prevalence of ANC is approximately 24% (14). In sub-Saharan Africa, utilization rates are 30% in urban areas and 6.9% in rural areas, significantly below WHO recommendations (15). A study in Ethiopia showed first-trimester scan rates of 5.2%, 3.7%, and 8.1% for the Amhara, Oromia, and SNNP regions, respectively, with second-trimester scans of 22.2%, 26.6%, and 48.6% respectively. Only one in six eligible women received a scan, which is much lower than the WHO's recommendation of at least one scan before 24 weeks of gestation (16).

Maternal and neonatal morbidity and mortality are critical health indicators (12). Globally, in 2017, an estimated 295,000 women died due to pregnancy and childbirth, 99% of whom died in LMICs where ANC quality is low (16, 17). It has also been reported that up to 37% of patients are potentially misdiagnosed. All the above could be reduced by incorporating U/S services in their care (12, 14, 16). In the Philippines, providing ultrasound during prenatal visits reduced maternal and neonatal deaths by 6.3% and 26.1%, respectively, and was cost-effective (18). Although antenatal ultrasound use did not affect mortality measures, there is evidence suggesting that ultrasound can confirm and improve patient management for both obstetric and non-obstetric conditions. A recent cluster-randomized trial found that use of ultrasound in rural health centers did not impact antenatal care attendance, facility delivery, maternal mortality, neonatal mortality and stillbirths (19). In contrary, a study conducted in three regions of Ethiopia found that introducing ultrasound services at the primary health care level, by mid-level health professionals, led to an increase in both antenatal and postnatal care utilization (20).

The use of ultrasound in developing countries is limited by several factors, such as culture, religion, illiteracy, attitude, accessibility and the high cost of ultrasound equipment; the fee of using ultrasound in a private clinic; the lack of trained sonographers or physicians; and the skill required to perform the examinations (15). Studies have suggested that the long interval between visits from 32 to 36 weeks could result in non-detection of intrauterine growth restriction and other problems that could arise and cause foetal death in the third trimester (21, 22). Moreover, poor utilization of ultrasound has indicated a high prevalence of adverse perinatal outcomes, such as ectopic pregnancies, abortions, congenital anomalies, fetal death, and increased maternal and neonatal morbidity and mortality (23). A systematic review in Ethiopia revealed a high incidence of neural tube defects (63.3% per 10,000), which contributes increased abortion rates (7, 24).

Ethiopia is working to improve maternal health care quality, reduce morbidity and mortality, and achieve sustainable development goals (16). The Ethiopian Ministry of Health aims to provide at least one ultrasound scan for all pregnant women before 24 weeks of gestation (25). The USAID Transform: Primary Health Care Activity provides 100 ultrasound machines to health centers and trains206 mid-level providers in ultrasound use (26).

Despite these efforts, maternal and neonatal morbidity and mortality remain high due to poor utilization of prenatal ultrasound scans (16). This study addresses new variables, such as maternal knowledge and attitudes toward obstetric ultrasound utilization, which previous studies did not cover. As far as my search is concerned, no published studies have assessed the proportion of prenatal ultrasound utilization in the South Wollo Zone. Therefore, this study aimed to determine the prevalence and associated factors of prenatal ultrasound among pregnant women attending ANC in South Wollo Zone Public Hospitals.

## 2 Methods and materials

### 2.1 Study design, area and period

A hospital based cross-sectional study was conducted at the South Wollo Zone Public Health Institution from December to February 2023. The town is found in the southern Wollo zone, southeastern Ethiopia, and is approximately 401 km away from Addis Ababa, the capital city of Ethiopia. The zone comprises 14 governmental hospitals (one comprehensive specialized hospital, four general hospitals, and nine primary hospitals) and four private hospitals. There are also 119 health centers and 450 health posts in the zone. All public and private hospitals provide all types of obstetric care including antenatal care, ultrasound services and outpatient and inpatient services. According to South Wollo Zone Health Bureau reports, the estimated number of antenatal admissions in South Wollo Zone Public Hospitals is 1800 women per month. The financial coverage of ANC services in Ethiopia is cost free and limited by the state. According to the 2017 Central Statistical Agency (CSA) of Ethiopia, the total estimated population of the South Wollo Zone is 3,086,132 from which these women accounts for almost half of the entire population. The language spoken in the zone was mainly Amharic.

### 2.2 Study design

An institution-based cross-sectional study was conducted.

### 2.3 Source population

All pregnant women were receiving antenatal care in South Wollo Zone Public Hospitals.

### 2.4 Study population

All pregnant women were receiving antenatal care at selected South Wollo Zone Public Hospitals during the data collection period.

### 2.5 Inclusion criteria

All pregnant women who attended antenatal care and who had been living for at least 6 months in the southern Wollo zone were included.

### 2.6 Exclusion criteria

Pregnant women who were severely ill and unable to communicate during the data collection period.

### 2.7 Sample size determination

The sample size was calculated based on a single population proportion formula using the following assumptions. The use of prenatal ultrasound.

Taken *p* = 50%.

$$n=\frac{{\left(z_{\alpha /2}\right)}^{2}\times p(1-p)}{d^{2}}$$

where *n* is the minimum sample size needed, *p* is the estimated proportion of prenatal ultrasound utilization, *z* is the standard value of the confidence level of *α* = 95% and *d* = 0.05 is the margin of error between the sample and the population.

For this study, *p* = 60.7%, since a study was performed in Jimma, Oromia Region, in Ethiopia on the proportion of prenatal ultrasound utilization (19).

$$p=0.607,\frac{z\alpha }{2}=1.96,d=\;0.05\frac{1.96{\;}^{2}\times 0.607\;\times (1-0.607)}{{\left(0.05\right)}^{2}}=357$$

Since we used two-stage sampling procedures and then multiplied by deff or 1.5, which is almost equal to 536, by adding a 10% nonresponse rate, the final sample size became **590**.

### 2.8 Sampling techniques and procedure

**Figure 1:**
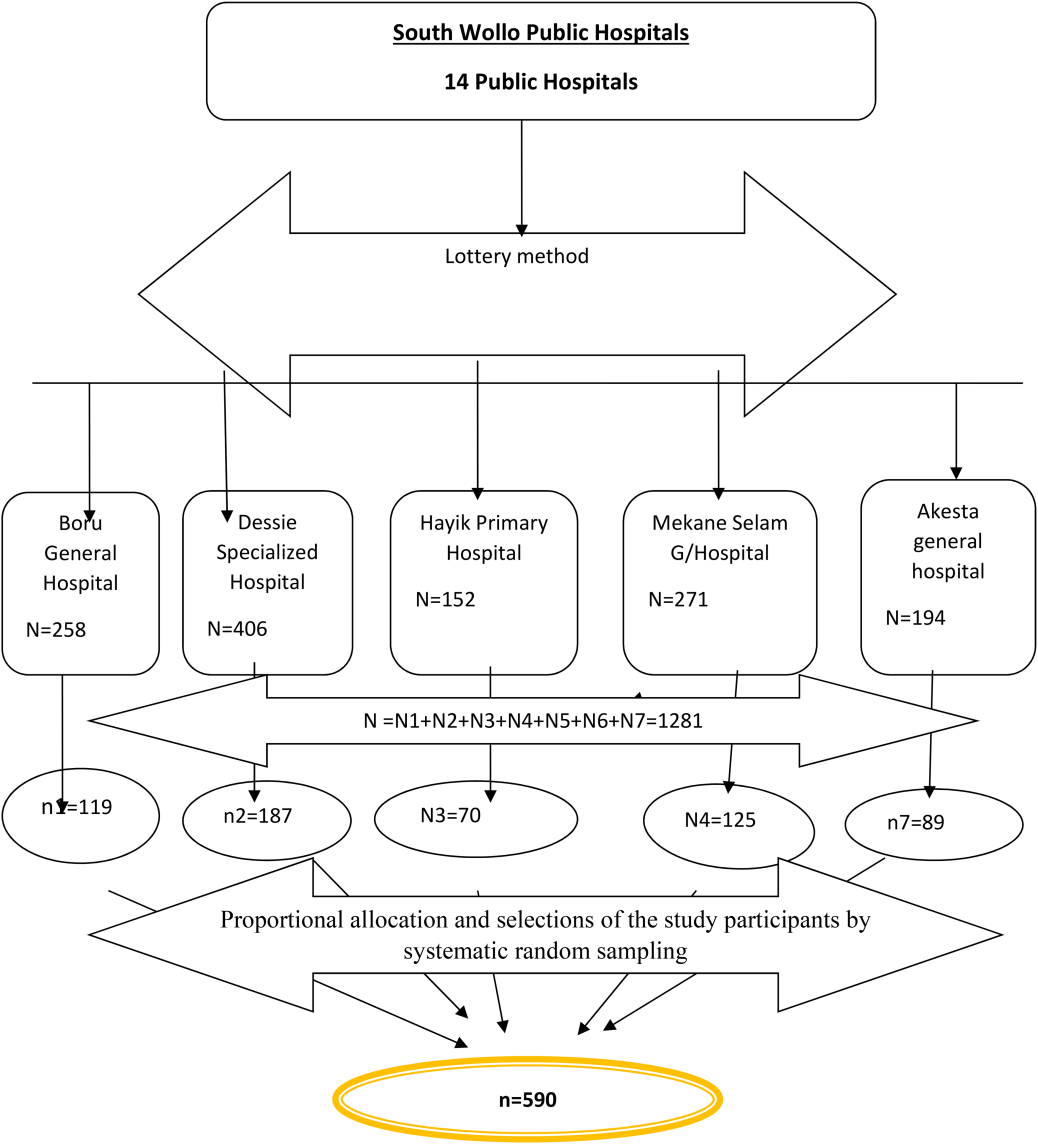
Schematic representation of sampling procedure for prenatal ultrasound utilization and associated factors among women who attending ANC in South Wollo Zone Public Hospitals, North-east, Ethiopia, 2023.

There are fourteen public hospitals in the South Wollo Zone. Of these, five public hospitals were selected randomly by the lottery method. The allocation of the samples to the hospitals was performed proportionally based on the average number of clients who received antenatal care at each hospital in the most recent 3-month report of each health facility. Study participants were selected at each facility by systematic random sampling techniques. The participants were selected in the order in which they came to health facilities. Participant card numbers were used to systematically select participants in every Kth, interval taking *K* = *N*/*n* = 974/590 = 1.65 ≈ 2. The first sample was selected randomly and then samples were taken every *K*^th^ interval until the required sample size was obtained. (where *N* is the source population taken from the Zone Health Bureau Three-month Antenatal Care Report of five selected hospitals and *n* is the sample size for this study (as shown in Figure 1).

### 2.9 Dependent Variables

Proportion of prenatal ultrasound utilization

### 2.10 Independent variables

• **Sociodemographic factors:** Maternal age, residence, ethnicity, marital status, monthly income, religion, educational level of the mother, and husband's education
• **The obstetric factors:** included parity, gravidity, history of abortion, history of ectopic pregnancy, history of recent congenital anomaly, illness experienced in recent pregnancy, and twin pregnancy
• **Maternal factors**: maternal knowledge of obstetric ultrasound findings and maternal attitudes toward obstetric ultrasound findings.

### 2.11 Operational definitions

**Obstetric ultrasound utilization**: In this study, the utilization of ultrasound by pregnant women was defined as obtaining ultrasound services at least once during a recent pregnancy (27).

**Knowledge of obstetric ultrasound**: Respondents who answered more than or equal to 6 questions (50%) of knowledge-related questions correctly provided were considered to have good knowledge. Those who answered fewer than 6 questions (<50%) were considered to have poor knowledge of obstetric ultrasound utilization (28).

**Attitude toward obstetric ultrasound**: The respondents whose responses “agree” to >50% of the questions regarding the attitude provided are categorized as having a good attitude toward obstetric ultrasound, whereas those whose responses “agree” to <50% disagree to the majority of questions are considered those with poor attitudes toward obstetric ultrasound (28, 29).

### 2.12 Data collections tool and procedures

The data were collected using semi structured interviewer-administered questionnaires. The questionnaire was prepared by reviewing different published literature and adapted to the objective of this study (25, 30–32), but knowledge and attitude assessment questionnaires were adopted from one study (28). The questionnaires were modified to suit the local conditions and it consisted of sections related to Socio-demographic characteristics, obstetric factors, maternal knowledge about obstetric ultrasound factors, and maternal attitudes towards obstetric ultrasound factors.

Five data collectors with BSc degrees in midwifery with two supervisors were recruited. The data collectors undertook face-to-face interviews using a semi structured and pretested Amharic questionnaire. Internal consistency/reliability of the item was checked by computing Cronbach's alpha. The value of Cronbach's alpha for knowledge assessment was 0.96 and attitude was 0.82.

### 2.13 Data quality control

The questionnaire was initially prepared in English. The English version was translated to the Amharic local language and translated back to English to ensure internal consistency by language experts. The quality of the data was ensured during collection, coding, entry, and analysis. Before the data were collected, one day of training and orientation were given to the data collectors and supervisors. Follow-up was also performed during the data collection. Moreover, the questionnaires were pretested on 29 participants (5% of the sample) at Woldia Hospital 15 days before the data collection to ensure the clarity, wording, and logical sequence of the questions. The necessary modifications were made. In addition, the supervisors and principal investigator supervised the whole activity of the data collection process and checked the completed questionnaires every day for completeness, and correctness, and necessary corrections were made in a timely manner.

### 2.14 Data processing and analysis

The data were edited and cleaned for inconsistencies by using Epidemiologic Data (Epid-data) 4.6 version software and then subjected to Statistical Package for Social Science version (SPSS) 26 (manufactured in April 2019; Armonk, Nework, United States) for data analysis. Descriptive statistics such as frequencies and cross-tabulations were performed. Graphical presentations such as pie charts were used to present the findings of the study. The information was presented in tables and figures. Bivariable and multivariable logistic regression analyses were performed to determine the associations between outcome and explanatory variables. Variables with a *P*-value <0.25 in the Bivariate analysis were used as the cutoff points for eligibility in the multivariate logistic regression model.

An effort was made to assess whether the necessary assumptions for the application of multivariable logistic regression were fulfilled. In this regard, the Hosmer and Lemeshow goodness-of-fit test yielded a large *p* value (*p* > 0.05) and the result was 0.589. Multicollinearity was checked to determine the linear correlation among the independent variables by using the variance inflation factor (VIF), Tolerance test and standard error. Variables with an inflation factor >10, standard error >2 and tolerance <0.1 were excluded from the multivariable analysis. The confounding effect were also checked.

Variables without collinearity were entered into a multivariable model. Only variables with a *p* value <0.05 were included in the final model. Finally, the AOR and, 95% CI were considered associated factors for utilizing prenatal obstetric ultrasound.

## 3 Results

### 3.1 Sociodemographic characteristics of the respondents

A total of 562 pregnant women participated in this study, for a response rate of 95.3%. The mean age of the study participants was 29.93 (SD ± 5.53) years, with a minimum of 18 years and a maximum of 45 years.

Three hundred twenty-four (56.2%) patients were living in urban areas. The majority of the respondents (70.3%) were married, and 296 (52.7%) of the respondents were housewives. The educational level of the respondents varied from illiterate to above a college diploma, with the majority (48.2%) being illiterate, as shown in Table 1.

**Table 1:** Sociodemographic history of pregnant mothers who attended ANC in selected South Wollo Zone Public Hospitals, Ethiopia, 2023 (*n* = 562).

Variable	Category	Frequency	Percentage
Age	15–19	7	1.4
	20–24	101	19.6
	25–29	156	30.2
	30–34	177	25.4
	>35	121	23.4
Marital status	Married	395	70.3
	Single	24	4.3
	Divorced	61	10.9
	Widowed	82	14.6
Residence	Urban	316	56.2
	Rural	246	43.8
Education	College diploma	107	19
	And above	77	13.7
	Secondary	107	19
	Primary	271	48.2
	Illiterate		
Husband education	College diploma and above	122	21.7
	Secondary	145	25.8
	Primary	146	26.0
	Illiterate	149	26.5
	Orthodox	283	50.4
Religion	Muslim	205	36.5
	Catholics	26	4.6
	Protestant	47	8.4
	Other	1	0.2
	Housewife	296	52.7
	Student	58	10.3
Occupation	Government employee	116	20.6
	Private employee	85	15.1
	Others	7	1.2
Ethinicity	Oromo	76	13.5
	Amhara	382	68
	Tigrie	65	11.6
	Guragie	35	6.2
	Others	4	0.7
Income per month (Ethiopian birr)	<3,000	168	29.8
	3,000–5,000	197	35.1
	>5,000	197	35.1

**Other religion**, adventist; **Others occupation**, merchant, shopkeeper, designer, house painter; **Other ethinicity**, afar.

### 3.2 Obstetric and maternal health service characteristics of pregnant women in selected South Wollo zone hospitals in Northeast Ethiopia, 2023

Four-hundred-ninety-nine (88.8%) of the respondents were multigravida, five hundred thirty nine (95.9%) initiated antenatal visits after six months, and 379 (67.4%) had four or more antenatal visits. One hundred seventy-four (31.0%) of the respondents had a pregnancy that experienced medical illness while one hundred fifty-four (27.4%) mothers had a pregnancy ended in abortion. Ninety-eight (17.4%) of the mothers delivered congenital anomalies (Table 2).

**Table 2:** Obstetric history of pregnant women who attended ANC in selected Southern Wollo Zone Public Hospitals, Ethiopia, 2023.

Variables	Frequency	Percentage
Category		
Gravidity (*n* = 562)		
Primigravida	63	11.2
Mulitgravida	499	88.8
Parity		
Primipara	104	18.5
Multipara	458	81.5
Previous abortion (*n* = 562)		
Yes	155	27.6
No	407	72.4
Hx ectopic Px9 (*n* = 562)		
Yes	51	9.1
No	511	90.9
Gestational age of current Px (*n* = 562)		
First TM	95	16.9
Second TM	382	68.0
Third TM	85	15.1
Time of ANC initiation for current pregnancy (*n* = 562)		
≤6 month	539	95.9
>6 month	23	4.1
Frequency of ANC visit		
For current pregnancy (*n* = 562)		
One	63	11.2
Two to three	120	21.4
Four or above	379	67.4
Mild to moderate illness experienced previous Px or this Px (*n* = 562)		
Yes	174	31.0
No	388	69.0
Hx congenital anomaly birth		
Yes	98	17.4
No	464	82.6
Recent delivery of baby (*n* = 562)		
Yes	467	83.1
No	95	16.9
Mode of delivery (*n* = 467)		
SVD	375	80.3
CS	92	19.7
Weight of recent birth (*n* = 467)		
<25,000 gm	92	19.7
2,500–4,000 gm	348	74.5
>4,000 gm	27	5.8

SVD, spontaneous vaginal delivery; CS, cesarean section; TM, trimester; Px, pregnancy.

The most reported component of knowledge reported by participants was helping to estimate gestational age which was reported by approximately 412 (73.3%) mothers. Estimating fetal weight was the second most reported importance of ultrasound, with 397 (70.6%) reporting it. The least common component of knowledge regarding obstetric ultrasound was determining the cord and placental position which was reported by only 310 (55.2%) particpants (Table 3).

**Table 3:** Knowledge component on obstetric ultrasound among pregnant women who attended ANC in selected South Wollo Zone Public Hospitals, Ethiopia, 2023.

Variables	Yes	Percentage
Knew the importance of ultrasound to confirm pregnancy	389	69.2
Knew the importance of ultrasound to determine fetal position	356	63.3
Knew the importance of ultrasound to determine cord and placental position	310	55.2
Knew the importance of ultrasound to determine the expected date of delivery	345	61.4
Knew the importance of ultrasound to detect any defect or Congenital abnormalities during pregnancy	350	62.3
Knew the importance of ultrasound to detect complications of pregnancy	351	62.5
Knew the importance of ultrasound to detect amniotic fluid	364	64.8
Knew the importance of ultrasound to detect any assess fetal wellbeing	366	65.1
Knew the importance of ultrasound helps to confirm	378	67.3
The Presence of multiple pregnancy		
Knew the importance of ultrasound to estimate fetal weight	397	70.6
Knew the importance of ultrasound to estimate gestational age	412	73.3
Knew the importance of at least one ultrasound before 24 weeks	392	69.8
Good knowledge	368	65.5
Poor knowledge	194	34.5

**Figure 2:**
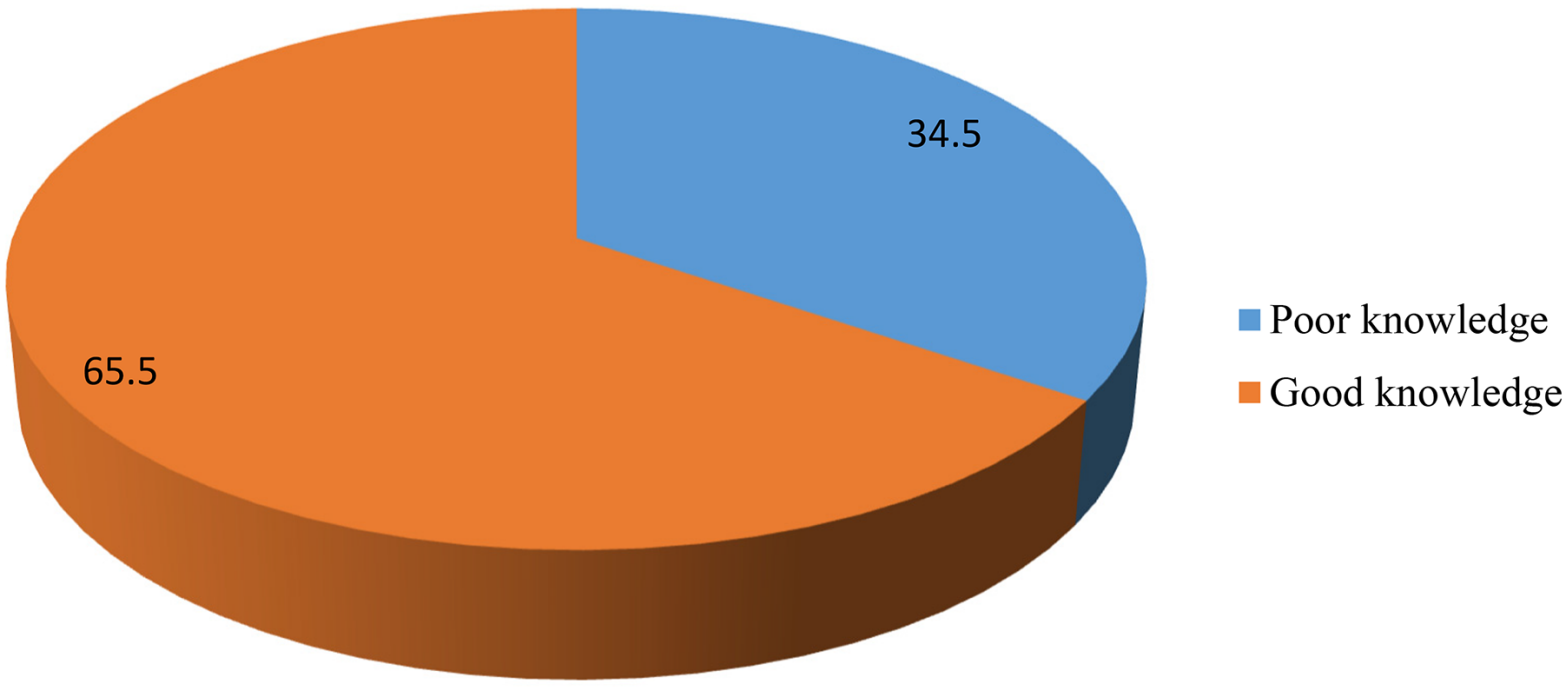
Knowledge of obstetric ultrasound finding among pregnant women attending ANC in selected South Wollo Zone Public Hospitals, 2023.

In this study, of 562 pregnant women, 368 (65.5%) had good knowledge of obstetrics ultrasound, while the remaining (194.34.5%) had poor knowledge of obstetric ultrasound. This indicates that more than half of the respondents were knowledgeable about the actual importance and effectiveness of obstetric ultrasound. The remaining women may to have a low rate of use of obstetric ultrasound or hinder women from being examined by ultrasound even in an emergency even though prenatal scanning improves pregnancy outcomes (as shown in Figure 2).

The most frequently mentioned component attitudes regarding obstetric ultrasound were “educating others about obstetric ultrasound is necessary”and“perceived that ultrasound is an essential investigation during pregnancy” (459; 81.7%). The second most mentioned component was “terminating a pregnancy if sex is other than you prefer,” where 437 (77.8%) respondents did not believe that terminating a pregnancy based on sex was the right decision (Table 4).

**Table 4:** Attitudes of pregnant women on obstetric ultrasound among pregnant women who attended ANC in selected South Wollo Zone Public Hospitals, Ethiopia, 2023.

Variables	Yes	Percentage
Perceived that obstetric ultrasound safe for the mother	409	72.8
Perceived that USS safe for the fetus	431	76.7
Perceived that USS lead to the anomaly	178	31.7
Perceived that USS is an essential investigation during Px.	459	81.7
Perceived terminating pregnancy if the sex of the child's other than you prefer, is right	437	77.8
Perceived that educating others about USS is necessary	459	81.7
Perceived that USS offer routinely	363	64.9
Negative attitude	249	44.3
Positive attitude	313	55.7

USS, ultrasonography.

**Figure 3:**
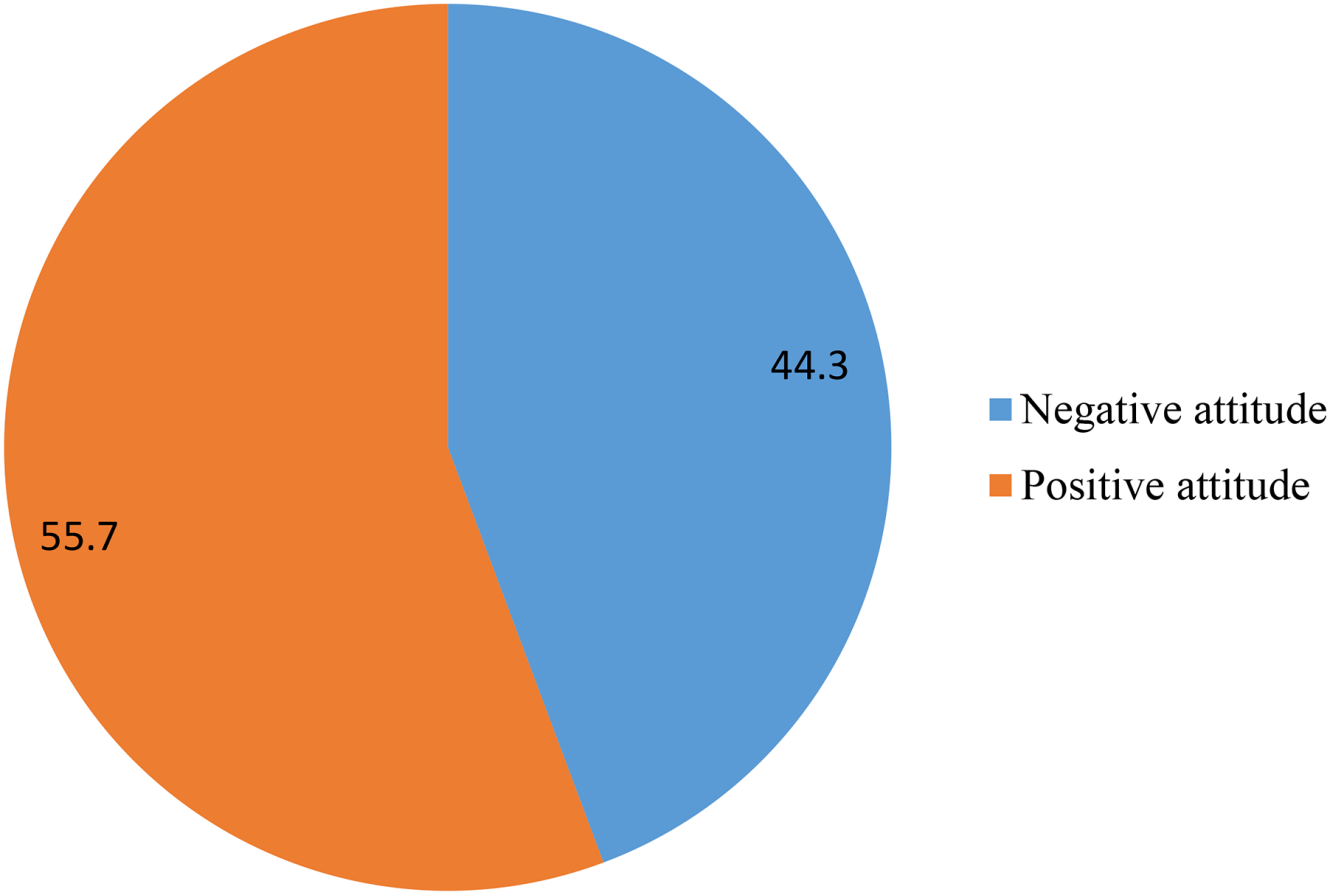
Overall attitudes among women who attended ANC in selected South Wollo Zone Public Hospitals, Ethiopia, 2023.

Out of 562 pregnant women, more than half (313, 55.7%) had a positive attitude toward obstetric ultrasound, while the remaining (249, 44.3%) had a negative attitude toward obstetric ultrasound. This indicated that nearly half of the women had a negative attitude toward prenatal ultrasound scanning. A negative attitude toward prenatal ultrasound scanning indicated that she was not willing to be scanned by obstetric ultrasound, which may have had a negative impact on the outcomes of pregnancy and motherhood (Figure 3).

### 3.3 Prenatal ultrasound utilization

Three hundred fifty-three (62.8%) respondents used prenatal ultrasound at least once during pregnancy, of whom 221 (62.5%) utilized it before 24 weeks of gestation and the remaining 122 (34.6) utilized it after 24 weeks of gestation. Nearly half, (51.3%) of the women requested the scan by themselves whereas 145 (41.1%) of the respondents were requested by clinicians (Table 5).

**Table 5:** Questions related to prenatal ultrasound utilization among pregnant women who attended ANC in the South Wollo Zone in the northeast region of Ethiopia, 2023.

Category		Frequency	Percentage
Prenatal OBS U/S utilization (*n* = 562)	Yes	355	62.8
	No	207	37.2
At what gestational age (*n* = 355)	<24 weeks	223	62.6
	>24 weeks	122	34.6
	Don’t remember	10	2.8
Who requested (*n* = 355)?	Clinician	145	40.8
	Herself	183	51.5
	Don’t remember	27	7.6
Reason not utilized Obs U/S (*n* = 207)	I think it is not nescceray.	19	9.1
	I fear it would cause injury to me and my baby.	62	30.6
	I didn’t have the information	36	17.2
	I cannot access it.	26	12.4
	Lack of money	64	30.7

### 3.4 Factor associated with the use of prenatal obstetric ultrasound

According to our bivariate logistic regression, fifteen variables were associated with the use of prenatal obstetric ultrasound: education status, husband's education status, residence, occupation, gravidity, parity, previous abortion, congenital anomaly delivery, the timing of ANC initiation, the frequency of ANC visits, recent delivery of baby, mode of delivery, recent birth weight delivery, knowledge of the woman and attitudes about the woman.

Respondents whose educational status was above college and diploma were nearly six times more likely to use prenatal ultrasound than did those with no formal education [crude odds ratio (COR) = 6.45; 95% CI: 3.56–11.72]. Similarly, pregnant women whose occupation was a government employee were more likely to utilize obstetric ultrasound than were those whose occupation was a housewife (COR = 7.99; 95% CI: 4.22–15.15).

However, according to our multivariate logistic regression analysis, only five variables were significantly associated with the use of obstetric ultrasound such as women's education status, women's knowledge, residence, occupation, and medical illness during pregnancy.

The odds of utilizing prenatal ultrasound among pregnant women who reside in urban areas were nearly five times greater than those who reside in rural areas, [adjusted odds ratio (AOR) = 4.82; 95% CI: 2.99–8.03]. Those respondents who encountered medical illness during pregnancy were nearly three times more likely to utilize prenatal ultrasound than were their counterparts (AOR = 3.03; 95% CI: 1.64–5.59). Similarly, women who had good knowledge of prenatal obstetric ultrasound were nearly seven times more likely to utilize prenatal ultrasound than were those who had poor knowledge (AOR = 7.32; 95% CI: 4.07–13.33) (see details in Table 6).

**Table 6:** Result of multivariate binary logistic regression analysis of factors associated with prenatal ultrasound utilization among pregnant women who attended ANC in selected South Wollo Zone Public Hospitals, Ethiopia, 2023.

Variables		Utilization of OU		COR (95% CI)	AOR (95% CI)
		YES (No.)	NO (No.)		
Residency	Urban	260	56	7.63 (5.19, 11.25)	4.82 (2.99, 8.03)**
	Rural	93	153	1.00	
	College	92	15	6.46 (3.56, 11.72)	2.41 (1.02, 5.70)*
Education	diploma above				
	Secondary	53	24	2.33 (1.36, 3.98)	3.55 (1.62, 7.82)*
	Primary	76	31	2.58 (1.60, 4.18)	2.10 (1.09, 4.05)*
	Unable to read and write	132	139	1.00	
Husband Education	College diploma above	96	26	2.10 (1.21, 3.63)	1.29 (0.56, 2.95)
	Secondary	95	50	1.08 (0.67, 1.74)	1.08 (0.67, 1.74)
	Primary	67	79	0.48 (0.30, 0.77)	0.37 (0.17, 0.68)*
	Unable to read and write	95	54	1.00	
Occupation	Housewife	35	22	1.00	
	Student	23	35	1.65 (0.34, 1.08)	0.95 (0.34, 1.64)
	Government employee	104	12	7.99 (4.22, 15.15)	4.05 (1.70, 9.64)*
	Private employee	67	18	3.43 (1.95, 6.06)	2.34 (1.58, 7.05)*
	Other	5		2.30 (0.44, 12.07)	2.74 (0.28, 26.88)
Gravidity	Multigravida	326	173	2.51 (1.47, 4.27)	1.77 (0.54, 0.83)
	Prim gravida	27	36	1.00	
Parity	Multipara	296	162	1.51 (0.98, 2.32)	0.47 (0.12, 1.87)
	Prim para	57	47	1.00	
Previous Abortion	Yes	126	29	3.45 (2.20, 5.40)	1.52 (0.78, 2.92)
	No	227	180	1.00	
Timing of ANC Intitition for current Px	≤6 month	346	193	4.10 (1.65, 10.13)	1.61 (0.48, 5.45)
	>6 month	20	161	1.00	
Frequency of ANC visit for Current Px.	One visit	24	39	1.00	
	Two to three visits	62	58	1.74 (0.93, 3.24)	0.55 (0.22, 1.37)
	Four visits and above	267	112	3.87 (2.22, 6.74)	0 0.88 (0.46, 2.66)
Gestational age for current Px.	First TM	58	37	1.00	
	Second TM	237	145	1.04 (0.65, 1.65)	1.35 (0.69, 2.64)
	Third TM	58	27	1.37 (0.74, 2.54)	1.50 (0.61, 2.67)
Mild to moderate Illness Experienced with this Px.	Yes	148	26	5.08 (3.20, 8.06)	3.03 (1.64, 5.59)*
	No	205	183	1.00	
Congenital anomaly delivery	Yes	76	22	2.33 (1.40, 3.88)	1.32 (0.64, 2.72)
	N	277	187	1.000	
Recent delivery of baby	Yes	299	168	1.35 (0.86, 2.12)	1.28 (0.36, 1.28)
	No	54	41		
Knowledge	Good knowledge	295	73	9.48 (6.35, 14.14)	7.36 (4.06, 13.32)**
	Good knowledge	58	136	1.00	
Attitude	Negative attitude	210	103	1.51 (1.07–2.13)	0.81 (0.46–1.45)
	Positive attitude	143	106	1.00	

* Statistically significant at *P* < 0.05.

** Statistically significant at *p* <0.001, 1 = reference category.

## 4 Discussion

This study was designed to assess the proportion of pregnant women utilizing prenatal ultrasound and associated factors mong those attending ANC in five public hospitals of South Wollo Zone. The proportion of prenatal ultrasound utilization in this study was 62.8% [95% CI: (58.7%–66.8%)]. This result was in line with the findings of prenatal ultrasound utilization described in Jimma Zone public hospitals in Ethiopia, which reported a 60.7% (33). This result was also consistent with the findings recorded in southeastern Nigeria, which were58.7% (34). However, the result of the current study were lower than tthose obtained in Uganda (35) and accordance with the local government Zaria, Kaduna State, northern Nigeria where the proportion of patients utilizing ultrasound was 83.5% (36).

These finding were also lower than those of a cluster rondomized study conducted in Eastern China (96.1%) (37). This variation may be due to socioeconomic factors, the propensity to use services and geographical differences between Ethiopia and Eastern China. The next step might be to focuses on both urban and rural pregnant women as China's study included only rural pregnant women. Moreover, the discrepancy might be due to variations in access to use services, barriers to use services, health service systems, and health policy programs.

In contrast, the percentages of patients in the current study was greater than that in a study conducted in Kenya (49.7%) (38). The difference may be atributed to variations in health policy strategies for child and maternal health care between Ethiopia and Kenya.

The findings of this study showed that there was a strong association between women's knowledge of prenatal obstetric ultrasound and prenatal ultrasound utilization. Pregnant women who had good knowledge were 7.36 times more likely to utilize prenatal ultrasound than pregnant women who had poor knowledge. This result was consistent with the findings recorded in Jimma, Ethiopia (21,33), and in different countries, e.g., a peri-urban health center in Uganda (35) and the main referral hospital, in Sokoto, Nigeria (27). Pregnant women with good obstetric ultrasound knowledge have a positive attitude toward obstetric ultrasound scans. The findings of this study align with research conducted in Gedeo Zone, Ethiopia (39). This indicates that increased awareness of obstetric ultrasound enhances its utilization. This, in turn, facilitates early detection and management of obstetric complications, ultimately contributing to reduced perinatal mortality. Greater knowledge fosters confidence in ultrasound's role in predicting pregnancy outcomes, whereas misconceptions may discourage its use. Obstetric ultrasound has become a critical tool in obstetrics, and its benefits have undeniably contributed to improved pregnancy outcomes (39–41). Therefore, ensuring that all antenatal women receive obstetric ultrasound scans is essential for the prevention and management of obstetric complications, ultimately leading to improved pregnancy outcomes, as recommended by the World Health Organization (WHO). To bridge gaps in knowledge and accessibility, obstetric care providers should integrate routine ultrasound assessments into maternity services while promoting education on its benefits, particularly for rural women with limited access. Strengthening maternal health policies and integrating educational interventions into antenatal care programs could further enhance uptake of ultrasound utilization, contributing to better maternal and neonatal outcomes across the countries.

In this study, pregnant mothers who reside in urban areas were more likely to utilize obstetric ultrasound than mothers who reside in rural areas (AOR = 4.82; 95% at CI: 2.99–8.03). This is probably due to the greater accessibility of information and awareness about prenatal ultrasound in urban areas than in rural areas and the variation in the distance of health facilities. Multiple sources of information about prenatal ultrasound data are obtained through television (TV) programs, private clinics, etc., for urban women rather than for rural pregnant women. Another reason may be the limited expansion of infrastructure in the rural areas compared to urban areas. These findings are in line with, other findings conducted in dilla (29). Therefore, the South Wollo Zone Health Office, in collaboration with nongovernmental organizations (NGOs), should prioritize the expansion and enhancement of healthcare services in rural areas through a multifaceted approach. This includes the construction of well-equipped health centers, the deployment of adequately trained healthcare professionals, the implementation of telemedicine services, and the introduction of mobile health units to extend services to remote communities. Furthermore, advocating for policies that ensure the equitable distribution of healthcare resources, including the provision of prenatal ultrasound services, across rural regions is essential for closing the urban-rural healthcare gap.

Medical illness during pregnancy was the third explanatory variable that was significantly associated with the use of prenatal ultrasound. Pregnant mothers who had medical illness during pregnancy were 3.03 times more likely to utilize prenatal ultrasound than were those who were free of illness during pregnancy. Similarly, a study from Canada, showed that medical illness during pregnancy was significantly associated with the use of prenatal ultrasound (42). This might be because of the perceived fear of losing their pregnancy and the complications associated with it. The authors suggest that policymakers and guideline developer's consider incorporating more frequent ultrasound scans into the antenatal care schedule for pregnant women with medical conditions, as this strategy can help mitigate concerns about pregnancy loss and related complications. Further more women with history of pregnancy loss or anomaly detection might feel that their pregnancy loss related to poor utiliztions of ultrasound services. Another study in India showed that medical conditions such as abdominal problems and urinary tract infections (UTIs) were among the most common conditions (12, 31). A possible explanation might be that abdominal pain and urinary tract infection during pregnancy may lead to frequent antenatal visits and subsequently greater opportunities for ultrasound scans. The more patients visit a health facility with complaints during pregnancy, the more scans they obtain.

In this study, occupation was also a predictor variable that was significantly associated with the use of prenatal ultrasound. The pregnant women whose occupations were government or private were 4.06 and 3.34 times more likely to utilize obstetric ultrasound, respectively, than were those whose occupations were housewives respectively. Similarly, in China, occupation was significantly associated with the use of prenatal ultrasound (12, 37). In contrast to these findings, a study performed in Nigeria reported that occupation does not affect the utilization of prenatal ultrasound (27). This may be explained by the differences in sociodemographic background and economic status the two countries. In this study, the educational level of the women was another predictor variable associated with the utilization of prenatal ultrasound. An education level above the primary level was significantly associated with prenatal ultrasound utilization with (OR = 2.10; 95% at CI: 1.09–4.05). This study proved that mothers with a high level of education had more requests for ultrasound scans, possibly because their awareness of pregnancy complications is greater than that of mothers with low education levels. Furthermore, families with high socioeconomic status had better access to sonography and did not mind financial factors, as previous studies have also supported these findings (29, 43).

Similar findings were obtained in a study conducted in Jimma (Ethiopia), in which individuals whose educational level was above primary level were more likely to undergo obstetric ultrasound than their counterparts were (33). This may be explained by the fact that the educational status of the pregnant mother increases, and exposure to pieces of information may also increase. Discussing and disseminating of this issue may become more common among educated pregnant women than among those younger primary school. The level of education tends to influence the methods by which women obtain and analyze information about ultrasound.

## 5 Strengths and limitations of the study

This research addressed pregnant women in different areas, both urban and rural by considering hospitals where antenatal flow is highest in all areas. The first study area in particular is the major strength of this study. Regarding the limitations of the current study, the literature on prenatal ultrasound utilization was limited, so discussing the findings of the study was difficult. While the study was designed as an institution-based cross-sectional study, it did not include women who received antenatal care at a private clinic. Moreover, The limitation of this study is, it is impossible to make causal inferences due to the cross-sectional nature of the study.

## 6 Conclusions

According to the current findings, the proportion of prenatal ultrasounds utilized was 62.8%, but the prevalence prenatal ultrasounds was still much lower than that recommended by FIGO and WHO, which suggest that all pregnancies should undergo a minimum of two scans throughout pregnancy. The educational status of the pregnant woman, occupations, knowledge, urban residency, and medical illness during pregnancy were significantly associated with the use of prenatal ultrasound. Therefore, the authors recommended for health care providers to educating mothers on the purposes of obstetric ultrasound and including a prenatal ultrasound screening as part of antenatal care is needed.

## 7 Recommendation

In light of these findings, the following recommendations are proposed:

The authors recommended for health care providers to educating mothers on the purposes of obstetric ultrasound and including a prenatal ultrasound screening as part of antenatal care is needed.
The authors suggest that policymakers and guideline developers consider incorporating more frequent ultrasound scans into the antenatal care schedule for pregnant women with medical conditions, as this strategy can help mitigate concerns about pregnancy loss and related complications
Authors recommended strengthening maternal health policies and integrating educational interventions into antenatal care programs could further enhance uptake of ultrasound utilization, contributing to better maternal and neonatal outcomes across the countries.
The south wollo zone health office, in collaboration with NGOs, should prioritize expanding rural healthcare services and task health extension workers with educating rural women about the importance of obstetric ultrasound as part of their routine duties.

## Data Availability

The original contributions presented in the study are included in the article/Supplementary Material, further inquiries can be directed to the corresponding author.
