# Supplementary material for: Prenatal ultrasound utilization and associated factors among pregnant women attending antenatal care in south Wollo zone public hospitals, north east, Ethiopia, 2023
Source: Front Digit Health. 2025 Apr 28;7:1547547. doi: 10.3389/fdgth.2025.1547547 (PMC12066484; doi:10.3389/fdgth.2025.1547547)
Supplement: Supplementary file 1 [file Datasheet1.pdf]

Supplementary Material

Supplementary Material- I

Supplementary Material I- Information sheet

Good morning/afternoon, my name is_________ and I am one of the data collector for the study being conducted by Belay susu, on a topic entitled "the prevalence of prenatal ultrasound utilization and associated factors among pregnant women attending ANC in South Wollo Zone Hospitals, North East Ethiopia, 2022''.

For this study, you are selected as a participant and before getting your consent to participate in the study, all the necessary information that you need to know related to the study is stated as follows;

Objective: To Assess Utilization of Prenatal Ultrasound and Associated Factors among Pregnant Women Attending Antenatal Care in South Wollo Zone Hospitals, North East Ethiopia, 2022.

Significance of the study: The rationale of this study is to increase the standard of pregnancy care and provide evidence about existing constraints with the prevalence and associated factors of prenatal obstetric ultrasound utilization. The result of this study can also help as baseline data or source of information for further study. Your cooperation and willingness to participate for the study is very helpful

Supplementary Material-II

English Version Questioners and Informed Consent Statement

Informed Consent Form

Hello! My name is ______________________________. Here, at Wollo University, College of Medicine and Health Science, School of Nursing and Midwifery, Department of Midwifery; I will be undertaking research aimed to assess the proportion of prenatal ultrasound utilization and associated factors in South Wollo Zone Hospital, North East Ethiopia, 2022.

You are kindly requested to participate in this study and provide the information required. Your participation in this study is completely voluntary bases and you have a right to refuse, to take part, or to stop giving information at any time. For your participation in the study, no payment will be granted or has no special privilege to you. Besides, you're not obligated to answer any question which you do not wish to answer. If you feel discomfort responding to any of the questions, please feel free to drop it any time you wish to do so. I assure you that your name will not be mentioned anywhere. Filling the questionnaire will take about 30 minutes. The information that you give me will be kept confidential and won't be accessible to a third party; only be used for the research purpose and burnt at the end of the survey.

Could I have your permission to continue?

1. Yes 2. No. Stop and thank the respondents.

Name & Signature of interviewer: _____________________ Date: ________________

Name & Signature of Supervisor: ______________________Date: _______________

Respondents ID number ....Health center……………………

Supplementary files

Table 1; Socio-demographic history of pregnant mothers attending ANC at South Wollo zone Public Hospitals North -east, Ethiopia from-to- 2022 G.C

Code	Variables	Responses	Remark
101	How old are you? (put age in years)	………………………………	
102	What is your marital status	1.Married<br>2.Single<br>3.Divorced<br>4.Widowed	
103	What is the highest grade you completed?	1. College diploma and above<br>2. Secondary3. Elementary4. Una	
104	What is the highest grade your husband completed?	1. College diploma and above<br>2. Tech. /voc. Certificate3. Secon	
105	Where do you live?	1.Urban<br>2.Rural	
106	If urban Q.No. 105.Duration of urban residence(Enter in years)	……………………………	
107	What is your Religion?	1.Orthodox<br>2. Muslim<br>3. Catholics<br>4. Protestant<br>5. Other	
108	What is your ethnicity?	1.Oromo<br>2. Amhara<br>3. Tigrie	

		4. Guragie<br>5. Others	
109	What is your occupation?	1.Housewife<br>2. Student<br>3. Government employee<br>4. Private employee<br>5. Others	
110	How much is your monthly income?(put in Ethiopian birr)	………….	

Table 2: Obstetric History of Pregnant Women who attend ANC atSouth WolloZone PublicHospitals North-east, Ethiopia, 2022.

Code	Variables	Responses	Remark
200	How many times have you ever been pregnant including this one? (put in number)	....	
201	How many times have you ever give birth?(put in number)	....	
202	Have you ever had a pregnancy that miscarried, aborted, or ended in a stillbirth?	1.Yes<br>2.No	
203	What is gestational age of current pregnancy?(put in exact month)	……………	
204	When did you initiate ANC?	……………..	
205	How many times did you receive antenatal care during last pregnancy?	…………….	

206	Have you had ectopic pregnancy?	1. yes<br>2. no	
207	Have you experience illness with recent or this pregnancy?	1.Yes<br>2.No	
208	Have you had delivery of baby with congenital malformation?	1. Yes<br>2. No	
209	If yes for Q 208, What was the mode of delivery for the recent child?	1.SVD<br>2.CS	Based on Q208 answer Q209 and Q210
210	Birth weight of the baby at birth?(put in exact weight in kg)	…………….	

Code	Variables	Responses	Remark
	Part III: knowledgerelated questions on prenatal ultrasound utilization among pregnant women who attend ANC at South Wollo Zone North-east, Ethiopia, 2022.		
300	Do you think ultrasound scan during pregnancy helps to confirm pregnancy?	Yes<br>No	
301	Do you think ultrasoundscan during pregnancy helps todetermine the fetal position?	Yes<br>No	
302	Do you think ultrasound scan during pregnancy helps to determine the	Yes<br>No	

	cord and placenta position?		
303	Do you think ultrasound scan during pregnancy helps to determine the expected date of delivery?	Yes<br>No	
304	Do you think ultrasound scan during pregnancy helps to detect any defect or congenital abnormalities during pregnancy?	Yes<br>No	
305	Do you think ultrasound scan during pregnancy helps to detect complication of pregnancy?	Yes<br>No	
306	Do you think ultrasound scan during pregnancy helps to detect amniotic fluid volume?	Yes<br>No	
307	Do you think ultrasound scan during pregnancy helps to assess Wellbeing of the Fetus?	Yes<br>No	
308	Do you think ultrasound scan during pregnancy helps to confirm presence of multiple pregnancies?	Yes<br>No	
309	Do you think U/S scan during		
309	Do you think ultrasound scan during pregnancy helps to estimate Weight fetal weight?	Yes<br>No	
310	Do you think ultrasound scan during pregnancy helps to estimate Gestational Age?	Yes<br>No	
311	Do you think every pregnant mother should have at least one U/S scan before 24 weeks of gestation?	1. Yes<br>2. No	

Table4: Attitude question on prenatal ultrasound utilization among pregnant women who attend ANC at South Wollo Zone North-east, Ethiopia, 2022.			
Code	Variables	Responses	Remark
400	Obstetric ultrasound is safe for the mother.	1. Strongly Disagree<br>2. Disagree<br>3. Not Sure<br>4. Agree<br>5. Strongly Agree	
401	Obstetrical ultrasound is safe for fetus	1. Strongly Disagree<br>2. Disagree<br>3. Not Sure<br>4. Agree<br>5. Strongly Agree	
402	Obstetric ultrasonography can lead to congenital anomaly.	1.Strongly Disagree<br>2.Disagree<br>3.Not Sure<br>4. Agree<br>5. Strongly Agree	
403	Obstetrical ultrasound in an essential investigation during pregnancy	1.Strongly Disagree<br>2.Disagree<br>3.Not Sure<br>4. Agree<br>5. Strongly Agree	
404	To know the sex of your child before birth is right	1.Strongly Disagree<br>2.Disagree<br>3.Not Sure<br>4. Agree<br>5. Strongly Agree	
405	Terminating the pregnancy if the sex	1.Strongly Disagree	

	of the child is other than you prefer is right decision	2.Disagree<br>3.Not Sure<br>4. Agree<br>5. Strongly Agree	
406	Educating others about obstetrical ultrasound is necessary	1.Strongly Disagree<br>2.Disagree<br>3.Not Sure<br>4. Agree<br>5. Strongly Agree	
407	Ultrasound needs to be offered routinely	1.Strongly Disagree<br>2.Disagree<br>3.Not Sure<br>4. Agree<br>5. Strongly Agree	

Table 5: Questions related to prenatal ultrasound utilization among pregnant women who attend ANC at South Wollo Zone North-east, Ethiopia, 2022.

Code	Variables	Responses	remark
500	Have you ever exposed to obstetric u/s scan?	1. yes<br>2. no	
501	If yes to question number 500, at what gestational age?	<24 weeks<br>≥24 weeks<br>Do not remember	
502	If yes to question number 500, Who request the ultrasound scan?	1. Clinician<br>2. Herself<br>3. Do not remember	
503	If No to question number 500, what was your reason?	I think it is not necessary<br>I fear it would have injury to me and my baby<br>I did not have the information	

		I can’t access it<br>Lack of money<br>Other(specify)________	

Thank you for your patience and cooperation for responding the question

Name of the data collectors____________________

Sign ______________________________________

Date _________________________
